# Clinical and Radiological Outcomes of Closed-Loop, Double Button, Coracoclavicular Fixation for Extralateral (Neer Type IIC) Fractures of the Distal Clavicle

**DOI:** 10.7759/cureus.25228

**Published:** 2022-05-22

**Authors:** Andreas Panagopoulos, Irini Tatani, Constantinos Kattou, Antonis Kouzelis, Kyprianos Kolios, Ioannis-Panagiotis Athinodorou, Zinon Kokkalis

**Affiliations:** 1 Orthopaedic Department, Medical School, University of Patras, Patras, GRC

**Keywords:** closed-loop double button, coracoclavicular stabilization, cho type iic, neer type iib, distal end clavicle fractures

## Abstract

Background: The distal end of the clavicle accounts for 10-28% of all clavicle fractures of which 52.8% are considered displaced and require internal fixation due to their high percentage of non-union. Numerous surgical techniques have been described for the well-known Neer types IIA, IIB, and V. Still, the literature is scarce for the rare “extralateral” (type IIC) fracture where the fracture line is located lateral to the completely torn coracoclavicular (CC) ligaments; such small fractures are sometimes not amenable for standard locking plate fixation.

Methods: We present a series of seven patients treated surgically with closed-looped double button CC stabilization via an open approach. There were four males and three females with a mean age of 31 years (range: 19-46 years). The mechanism of injury was a motor vehicle accident in four cases and a fall from height in three cases. The average time from injury to surgery was 2.7 ± 1.3 days and the average follow-up period was 25.7 months (range: 16-48 months). A custom-made, closed-looped, double button device was made using the ProCinch Adjustable Cortical Fixation for anterior cruciate ligament (ACL) (Stryker, Kalamazoo, Michigan) and another standard or slotted button. The fracture was reduced anatomically and the device was tightened and secured with five to six knots. In two cases, additional interfragmentary sutures were used for extra stability. Postoperatively, the arm was immobilized in a simple sling for four weeks; passive assisted elevation up to 90 degrees was allowed from the second postoperative week, followed by active elevation after the sixth postoperative week. Radiological outcomes (bony union, loss of reduction, implant mispositioning, or subsidence of buttons) were assessed using serial plain radiographs. The Constant score (CS) and the Acromioclavicular Joint Instability Score (AJIS) were used for the final clinical evaluation, at least one year postoperatively.

Results: Bony union was achieved in all patients at a mean time of 2.7 months (range: 2.5-3.6 months). No cases of delayed union, loss of reduction, button migration, or subsidence were noted. The mean CS was 96.6 ± 3.4 and the mean AJIS score was 94.1 ± 4.7 in a mean follow-up period of 25.7 months (range: 16-48 months). One patient developed a hypertrophic scar and another had mild skin irritation by the suture knots; no other complications were noted except for one patient who developed an early superficial skin infection managed with antibiotics and debridement under local anesthesia. Four patients who participated in sports before injury were able to regain almost full activity seven to nine months after the operation. All were satisfied with the final result. Two patients showed ossification of the CC ligaments with no significant clinical implications.

Conclusions: Although we retrospectively reviewed a small series of patients, we were able to demonstrate a complete rate of fracture union and excellent clinical outcomes with no major complications. Type IIC distal clavicle fractures are rare and require special attention in terms of reduction and optimal fixation. Open CC fixation with closed-looped double buttons is a relatively easy and reproducible technique. We advocate the readjustment of Neer’s classification, including “extralateral” fractures as a IIC subtype.

## Introduction

Clavicle fractures represent 2.6-4% of all adult fractures and occur most frequently in men under the age of 30 and above 60 years [1,2]. The distal end of the clavicle accounts for 10-28% of all clavicle fractures of which 52.8% are considered displaced and manageable operatively [2,3]. Forces are applied to the clavicle either as a result of a direct fall on the shoulder or onto an extended hand; motor vehicle accidents and sports injuries are the most common mechanisms of injury [4]. According to Craig’s modification [5] of the classic Neer’s classification, there are five types of distal clavicle fractures: the original Neer types I-III and the types VI (epiphyseal fractures in children) and V (avulsed inferior cortical fragment attached to the coracoclavicular (CC) ligaments). Type II fractures involve the CC ligaments with subtype IIA located medially to them and subtype IIB between them. However, this classification does not include the small-sized fractures lateral to the torn CC ligaments with an associated displacement of the medial clavicle (“extralateral” type) [6,7]. In a systematic review by Cho et al. [7], a new modified classification system was proposed, which includes subtype IIC and IID fractures. The former is described as a fracture lateral to the CC ligaments, with both being torn from the medial fragment (“extra-lateral type”). The latter involves a comminuted fracture, with the CC ligaments remaining intact and attached to the inferior fragment, thus representing the type V fracture in Craig’s classification.

There is no optimal surgical technique for managing unstable distal clavicular fractures (types IIA, IIB, and V). Several fixation techniques have been proposed that are generally categorized as rigid (hook or locking plates, CC screws, and transacromial pins or Kirschner wires (KW)) and flexible (CC sutures, tapes, wires, anchors, or cortical buttons, with or without arthroscopic assistance) [8-12]. Combined techniques have also been proposed especially in terms of CC augmentation to a standard locking plate [13,14]. Despite this plethora of surgical treatment options, no single technique has been proven so far superior to another. The evidence is even more scarce for type IIC fractures, where the small lateral clavicle fragment is not always amenable to hold traditional hardware. Levy et al. [15] were the first to report on fractures classified as type IIC; out of 48 patients enrolled in the study, 30 were treated with simple CC suture stabilization and the rest underwent plate fixation and CC augmentation concluding that there is a need for modifying the original Neer’s classification. The purpose of this study was to present the clinical and radiological outcomes in a series of seven patients with extralateral (type IIC) fractures of their distal clavicle treated with open CC stabilization using a closed-loop double-button fixation.

## Materials and methods

We present a retrospective case series of seven consecutive patients (four males and three females; mean age: 31 years old; range: 19-46 years) who were treated at our university hospital between 2016 and 2020 for an “extra-lateral” type IIC fracture of their distal clavicle (Table 1).

**Table 1:** Demographics, mechanism of injury, clinical outcome, and complications of the included patients. CC: coracoclavicular; F: female; M: male; R: right; L: left; m: months; CS: Constant score; AJIS: Acromioclavicular Joint Instability Score.

Patient	Sex	Age	Side	Mechanism	Sport	Surgical technique	Follow up (m)	CS	AJIS	Complications
1	F	29	L	Motor vehicle accident	Basketball	Closed-loop double CC button	19	95	90	-
2	M	19	R	Fall from bicycle	Cycling	Closed-loop double CC button and additional transosseous sutures	20	98	91	Skin irritation (knots)
3	M	20	L	Fall from motorbike	Football	Closed-loop double CC button	17	100	96	Hypertrophic scar
4	Μ	46	R	Fall from bicycle	-	Closed-loop double CC button and additional transosseous sutures	16	90	92	Superficial infection
5	F	30	R	Fall from height	Squash	Closed-loop double CC button	48	95	96	-
6	M	35	L	Motor vehicle accident	-	Closed-loop double CC button	36	98	98	-
7	F	38	L	Fall from height	-	Closed-loop double CC button	24	100	96	-

**Figure 1:**
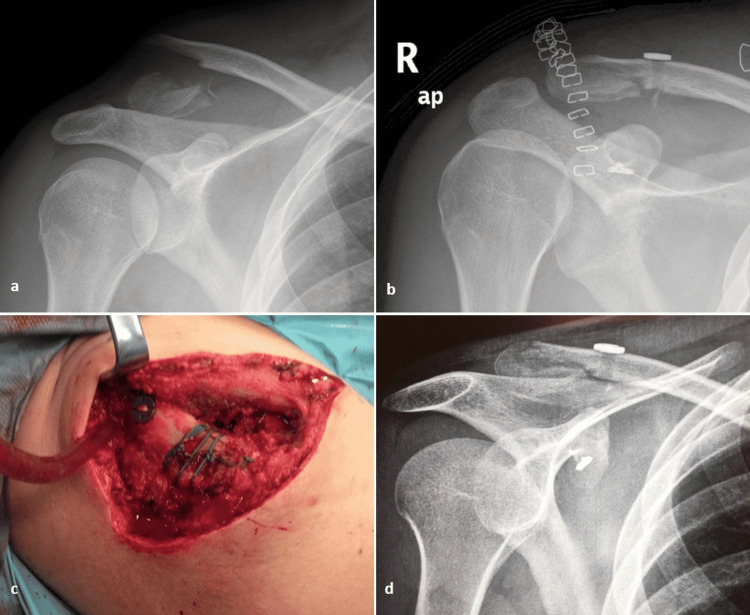
Radiological evaluation and intraoperative photo of patient 2. (a) Preoperative radiograph of an extralateral distal clavicle fracture, with comminution and marked vertical displacement. (b, c) Postoperative radiograph and intraoperative photo showing reduction of the fracture and additional interfragmentary suture repair in respect. (d) Final follow-up radiograph at six months showing maintenance of reduction.

Institutional ethical approval was obtained from the Institutional Review Board of Patras University Hospital (AΠ 231/10-11-2021) and all patients consented to participate in the study. The mechanism of injury was a motor vehicle accident in four cases and a fall from height in three cases; no other concomitant injuries were noted. All patients underwent surgery within one week of initial injury, with an average time from injury to surgery of 2.7 days (range: two to five days). The average follow-up period was 25.7 months (range: 16-48 months). All patients were operated under general anesthesia in a beach-chair position via a 6-cm vertical “strap” incision based on the coracoid process. A full-thickness incision to the deltotrapezial fascia was made for fracture exposure followed by blunt anterior deltoid splitting to prepare the base of the coracoid process. The coracoid tunnel was made first with a 4.5 mm cannulated drill, taking care of its accurate location, i.e., in the center of the coracoid and as close as possible to its base. The clavicular tunnel was made thereafter with the same cannulated drill, located 1.5-2 cm medial to the fracture site, trying to reproduce the anatomic location of the CC ligaments. A custom-made, closed-looped, double button device was made using the ProCinch Adjustable Cortical Fixation for anterior cruciate ligament (ACL) (Stryker, Kalamazoo, Michigan) and another standard or slotted button (G-Lok No Loop, Stryker, Kalamazoo, Michigan). The fracture was reduced by pulling the button sutures upwards and watching the gradual reduction of the fracture to its anatomical position; sometimes reduction forceps can be applied at the tip of the coracoid and the medial clavicle to facilitate that. The device was then tightened and secured with five to six knots. In two cases with oblique fracture extension, additional interfragmentary sutures (No. 2 Ethibond; Ethicon, Somerville, New Jersey) were placed for extra stability and interfragmentary compression (Figure 1).

All patients received two doses (i.e., one before surgery and one 12 hours later) of perioperative antibiotic treatment (IV cefuroxime) for infection prophylaxis and they were discharged the following day without any instructions for postoperative anticoagulation therapy. Postoperatively, the arm was immobilized in a simple sling for four weeks; passive assisted elevation up to 90 degrees was allowed from the second postoperative week, followed by active elevation after the sixth postoperative week. Sporting activities were allowed after the sixth postoperative month. Radiological outcomes (bony union, loss of reduction, implant mispositioning, or subsidence of buttons) were assessed using serial plain anteroposterior and/or Zanca or Alexander stress radiographs. The Greek translation of the Constant score (CS) [16] and the Acromioclavicular Joint Instability Score (AJIS) were used for the final clinical evaluation, at least one year postoperatively. In this study, non-parametric statistical tests were applied. Paired median comparisons were executed with Wilcoxon signed-rank test. To perform median comparisons of more than two distributions, the Kruskal-Wallis test was applied. All statistical tests were considered two-sided, and statistical significance was considered when p < 0.05. The statistical analysis was performed using R software for statistical computing (R Foundation for Statistical Computing, Vienna, Austria), along with the RStudio (Boston, Massachusetts) interface (both open-source products).

## Results

**Figure 2:**
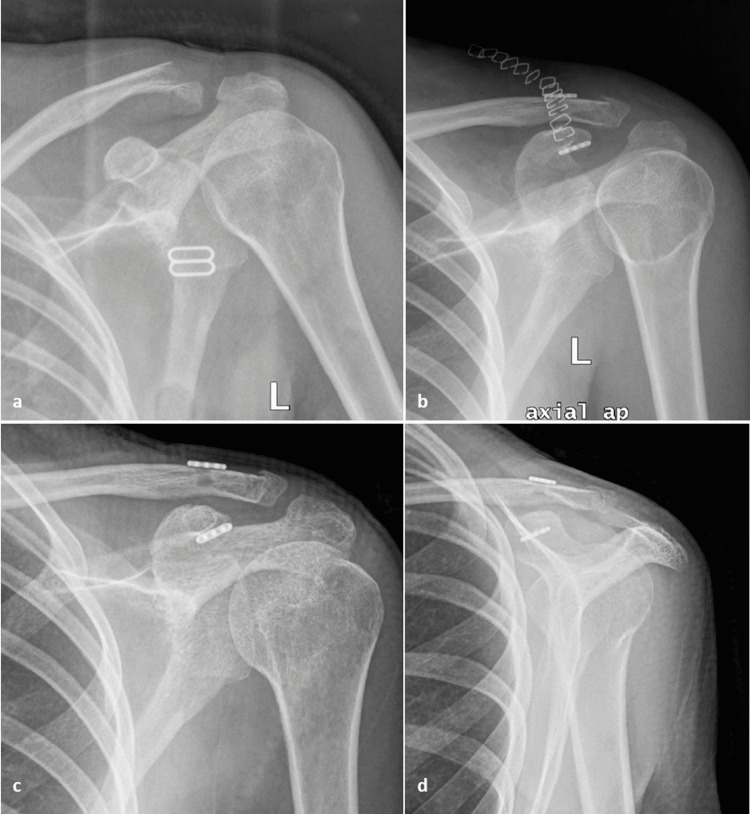
Radiological evaluation of patient 1. (a) Preoperative anteroposterior radiograph showing an extralateral fracture of the distal clavicle. (b) Excellent postoperative reduction using a closed-loop double button system. (c, d) Last follow-up radiographs (anteroposterior and Alexander view), 12 months postoperatively, showing maintenance of reduction and no signs of osteolysis or loosening.

Bony union at the fracture site was achieved in all patients at a mean time of 2.7 ± 0.6 months (range: 2.5-3.6 months). No cases of delayed union, loss of reduction, button migration, or subsidence were noted (Figure 2).

**Figure 3:**
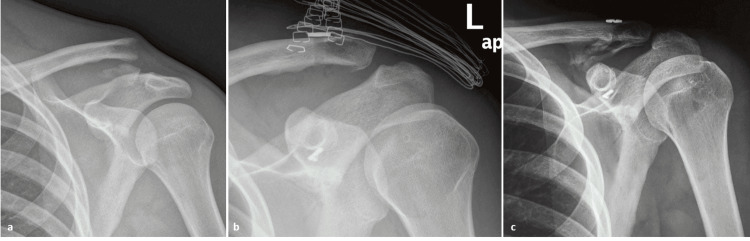
Radiological evaluation of patient 5. (a) Preoperative Zanca radiograph of an extralateral distal clavicle fracture with marked medial fragment displacement. (b) Postoperative Zanca radiograph showing excellent reduction of the fragment. (c) Final follow-up Zanca radiograph showing maintenance of reduction and calcification of coracoclavicular ligaments, a sign of adequate healing. The patient had excellent outcome scores.

Three patients with a confirmed union in their early X-rays refused to undergo the final radiological examination, although they came for the clinical evaluation. The mean CS was 96.6 ± 3.4 and the mean AJIS score was 94.1 ± 4.7 in a mean follow-up period of 25.7 months (range: 16-48 months). No statistical difference was found between scores at the final follow-up (p = 0.832). One patient developed a hypertrophic scar and another had mild irritation by the suture knots; no other complications were noted except for one patient who developed an early superficial skin infection managed with antibiotics and debridement under local anesthesia. Four patients who participated in sports before injury were able to regain almost full activity seven to nine months after the operation. The other three patients did not participate in sports and had sedentary occupations. All were satisfied with the final result. Two patients showed ossification of the CC ligaments with no clinical implications (Figure 3).

## Discussion

There is no optimal operative technique for the management of unstable distal clavicle fractures of types IIA, IIB/(IIC), and V/(IID). The surgical procedures that can be used for these types of distal clavicular fractures can be grouped into five main categories: (1) hook plate; (2) anatomic locking plates ± CC augmentation; (3) open or arthroscopic-assisted CC fixation (buttons, sutures, tapes, wires, and cables); (4) interfragmentary fixation; and (5) transacromial fixation with KW and/or tension band [2,3,8-15].

Oh et al. [8] published in 2011 a systematic review of 365 surgically treated Neer type II fractures and demonstrated a higher complication rate of the hook plate (40.7%) and tension band wiring (20.0%) in contrast to CC (4.8%), intramedullary (2.4%), and interfragmentary fixation (6.3%). Stegeman et al. [10] reported a meta-analysis of 21 studies in 2013, including 350 patients with Neer type II distal clavicle fractures, and found similar functional outcomes among the various treatment modalities (hook plate, standard plates, CC sutures, screws, and Knowles pins) but also an 11-fold and 24-fold increased risk of major complications for the hook plate compared to intramedullary fixation and suture anchoring in respect.

Boonard et al. [17] performed a systematic review and network meta-analysis of 547 patients in 2018 and found that Constant-Murley scores of CC fixation were significantly higher than those of hook plate whereas Asadollahi and Bucknill (2019) [9], in a systematic review of 11 comparative studies, including 634 patients, found no significant difference between the functional outcome and union rates but a higher complication rate of hook plate fixation in contrast to CC stabilization and the locking plates. Panagopoulos et al. (2021) [18] systematically reviewed the safety and efficacy of CC stabilization techniques, particularly in Neer type IIB/IIC fractures, and found very good to excellent clinical scores and overall major and minor complication rates of 2.6% and 12.8%, respectively.

A recent systematic review by Uittenbogaard et al. (2021) [19] in 2284 patients, including all Neer type II fractures (IIA and IIB), showed lower CS of the hook plate in comparison to CC fixation and also better clinical results when a standard locking plate was augmented with CC fixation; the latter conjecture was not confirmed in the most recent systematic review and meta-analysis (2022) conducted by Panagopoulos et al. [12] in our department. Specifically, this systematic review on the best available treatment options for Neer type IIB/IIC distal clavicle fractures showed not only a low level of evidence and heterogeneity among studies but also inconsistent methods of classification, inappropriate outcome evaluation, and underreporting of complications. Nevertheless, hook plate fixation showed again inferior clinical results with a high rate of minor complications but no worse rate of major complications. The best results were achieved by using either a locking plate fixation (with or without cortical button augmentation) or open CC stabilization techniques. Levy et al. (15) compared these two techniques (plate and open CC suture stabilization) in the unique type IIC fracture and found better results with the CC stabilization. The superiority of open CC fixation techniques has been recently demonstrated by both Malik et al. [20] and Yagnik et al. [11] who systematically reviewed the arthroscopic-assisted CC stabilization techniques; despite the overall good to excellent shoulder function in both reports, the first one found lower union rates of up to 70% and overall complication rates as high as 28.6%, and the second reported overall complication rates of 27.4%.

In the last decade, approximately 80% of the reported IIB distal clavicle fractures have been managed with CC stabilization techniques or locking plates [12]. For the rare subtype IIC, where the lateral fragment is relatively small, even a special distal clavicular plate may not be the ideal implant as has been recently noted in the comparative study of Levy et al. [15]. Kim et al. [6] and Cho et al. [7] proposed not only a new classification system to include the “extra-lateral” types but also an evidence-based treatment algorithm where subtypes IIA, IIB, and IID can be managed using locking plates, while for the subtype IIC, the authors recommend the use of CC fixation, as we did in our case series.

## Conclusions

Although we presented a small series of patients, we were able to demonstrate a complete rate of fracture union and excellent clinical outcomes with no major complications. Type IIC distal clavicle fractures are rare and require special attention in terms of reduction and optimal fixation. Open CC fixation with closed-looped double buttons is a relatively easy and reproducible technique. A modification of the existing Neer classification is essential to include this rare type of fracture.
